# Optimizing internet-delivered cognitive behaviour therapy for alcohol misuse: a study protocol for a randomized factorial trial examining the effects of a pre-treatment assessment interview and health educator guidance

**DOI:** 10.1186/s12888-020-02506-2

**Published:** 2020-03-17

**Authors:** Christopher Sundström, Heather Hadjistavropoulos, Andrew Wilhelms, Matt Keough, Michael Schaub

**Affiliations:** 1 Department of Psychology, University of Regina, 3737 Wascana Parkway, Regina, Saskatchewan S4S 0A2 Canada; 2 Department of Clinical Neuroscience, Karolinska Institutet, Stockholm, Sweden; 3 Department of Psychology, York University, Toronto, Ontario Canada; 4 Swiss Research Institute for Public Health and Addiction, University of Zurich, Zurich, Switzerland

**Keywords:** Alcohol, Internet interventions, Cognitive behavior therapy, Guidance, Assessment reactivity

## Abstract

**Background:**

Alcohol misuse is a common, disabling, and costly issue worldwide, but the vast majority of people with alcohol misuse never access treatment for varying reasons. Internet-delivered cognitive behaviour therapy (ICBT) may be an attractive treatment alternative for individuals with alcohol misuse who are reluctant to seek help due to stigma, or who live in rural communities with little access to face-to-face treatment. With the growing development of ICBT treatment clinics, investigating ways to optimize its delivery within routine clinic settings becomes a crucial avenue of research. Some studies in the alcohol treatment literature suggest that assessment interviews conducted pre-treatment may improve short- and long-term drinking outcomes but no experimental evaluation of this has been conducted. Further, research on internet interventions for alcohol misuse suggests that guidance from a therapist or coach improves outcomes, but more research on the benefits of guidance in ICBT is still needed.

**Methods:**

This study is a 2X2 factorial randomized controlled trial where all of the expected 300 participants receive access to the Alcohol Change Course, an eight-week ICBT program. A comprehensive pre-treatment assessment interview represents factor 1, and guidance from a health educator represents factor 2. All participants will be asked to respond to measures at screening, pre-treatment, mid-treatment, post-treatment and 3, 6 and 12 months after treatment completion.

**Discussion:**

This study will provide valuable information on optimization of ICBT for alcohol misuse within routine clinic settings.

**Trial registration:**

ClinicalTrials.gov, registered June 13th 2019, NCT03984786

## Background

Alcohol contributes to about 4% of the global burden of disease [1] and accounts for approximately three million deaths globally each year [2]. Common causes of illness, harm and death due to alcohol use include falls and accidents, cancers, liver disease and heart disease [3]. About half of all alcohol-related harm is caused by people with alcohol use disorders [4], but only around one in five of those who misuse alcohol are estimated to seek help within routine care [5, 6]. Further, most people who come to treatment, do so at a very late stage, usually after having experienced problems for at least a decade [7]. There are various psychological factors that inhibit or delay treatment-seeking among people with alcohol misuse [8]. At an early stage, people may struggle with problem recognition. They may be in denial of their problem, they may minimize or rationalize the consequences of their alcohol consumption or they may not believe that treatment will be effective. At a later stage, people may be fully aware of their need for help but they may still be deterred to seek treatment due to the severe and well-documented stigma associated with alcohol misuse [9]. Treatment alternatives for people with alcohol misuse that could bypass some of the factors that inhibit or delay treatment-seeking are sorely needed, and would provide an important addition to current treatment options.

### Internet-delivered cognitive behavior therapy for alcohol misuse

Internet-delivered cognitive behaviour therapy (ICBT) is a treatment alternative that consists of modules based on evidence-based cognitive behavior therapy delivered online [10]. A large number of studies on ICBT for depression and anxiety have been conducted over the past 20 years showing significant effects [11, 12], and as a result this form of treatment is now being implemented into routine care in countries around the world [13]. Alcohol misuse is the most stigmatized of all psychiatric conditions [14], and for this reason the anonymous nature of ICBT may be particularly suited for this population, while also being a valid evidence-based treatment alternative for people who live in rural areas where patients may need to travel significant distances to treatment clinics. ICBT for alcohol misuse is based on well-known, validated therapeutic approaches to treating substance use such as Relapse prevention [15] and Community Reinforcement Approach [16]. Most often, these programs consist of a number of modules where the user sets a drinking goal, identifies risk situations and learns to deal with cravings, social situations and relapse [17–19].

#### Guidance in ICBT for alcohol misuse

ICBT can be either self-guided, allowing users to complete modules by themselves, or guided, in the form of emails or online messages provided by a therapist or coach [20]. A consistent finding in research on ICBT for depression and anxiety is that guided ICBT tends to lead to greater effects than self-guided ICBT [21, 22], but education of the guide does not seem to have effect on strength of outcomes [22]. The literature on guided ICBT for alcohol misuse, however, is still sparse. A recent individual patient data meta-analysis found guided internet interventions for alcohol misuse to be superior to unguided ones [23]. However, the analysis did not distinguish between ICBT and other kinds of internet interventions, such as brief interventions. Two studies have compared guided ICBT to unguided ICBT finding small and medium differential effects sizes respectively [24, 25], while one study compared guided ICBT to a waitlist and found a large differential effect size [26]. Although these studies indicate that guidance is of benefit for this population, two of these studies only presented post-treatment follow-ups [25, 26], while the third presented a 6 month follow-up [24]. Thus, research is needed both to replicate this finding within routine care, and to investigate long-term outcomes of guidance in ICBT for alcohol misuse [27].

### Assessment reactivity in alcohol misuse trials

Research trials that aim to evaluate treatment usually entail some degree of assessment whereby participants respond to more or less structured questions about some aspect of their behaviour. Reporting on one’s own behaviour in this way may prompt change of that behaviour, a phenomenon which has been referred to as assessment reactivity (AR) [28]. Research suggests that AR may be beneficial in alcohol trials, as assessment appears to be associated with a subsequent change in drinking behaviour and alcohol-related problems [29]. Although the mechanisms by which assessment can change subsequent drinking are poorly understood, one hypothesis is that being asked to reflect on one’s drinking leads to a greater awareness of problem severity, which, in turn, leads to initiation or strengthening of motivation to change [30]. The clinical improvements related to AR were highlighted in a 2012 literature review [31] in which several studies investigated the effects of AR during follow-up. For example, in one factorial trial conducted in an outpatient substance use clinic, participants were randomized to receive follow-up interviews that were either frequent/infrequent (factor 1) and brief/comprehensive (factor 2). Results showed that those receiving brief and infrequent follow-up interviews had significantly higher alcohol consumption at the 12-month follow-up compared to other groups, suggesting a presence of AR [32]. In a second paper from the same trial, the researchers found that the groups who received the comprehensive and frequent follow-up interviews displayed significantly greater treatment engagement [33]. Other studies have focused on the effects of AR induced by pre-treatment assessments. In one study, females with alcohol problems received three assessments prior to being randomized to receive either individual or couples CBT. A secondary analysis of this data showed that 44% of participants became abstinent prior to treatment, and these individuals had significantly better drinking outcomes both during treatment and at 12-month follow-up [30]. The same pattern of results has been found in a trial aimed at adolescents with substance use problems, where all participants received an intake assessment before treatment start. By the first session, 51% had become abstinent, and further analysis showed that these adolescents had significantly better drinking outcomes at last session of the treatment than those not abstinent by first session [34]. Although these studies are correlational and therefore do not allow for causal interpretations, they imply that AR induced by pre-treatment assessment may lead to immediate reductions in alcohol consumption at the start of treatment and also that they may be related to reductions in alcohol consumption in the longer term. AR induced by pre-treatment assessment may thus be of great clinical value, rendering it worthy of exploration in clinical practice settings.

### Purpose and aims

The purpose of this study protocol is to present the rationale and methods of the Alcohol Change Course trial – a 2X2 factorial randomized controlled trial for people with alcohol misuse designed to examine optimal delivery of ICBT for alcohol misuse in a routine online therapy clinic. All participants in the trial will receive the Alcohol Change Course, an ICBT program for people with alcohol misuse, and the trial aims to evaluate the added and interaction effects of a pre-treatment assessment interview (factor 1) and health educator guidance (factor 2). We hypothesize that participants receiving a pre-treatment assessment interview will reduce their alcohol consumption more than those not receiving such an assessment interview at the 3 month follow-up (primary endpoint), and that participants receiving health educator guidance will reduce their alcohol consumption more than those not receiving such guidance at the 3 month follow-up. We will also conduct a post-treatment follow-up to assess treatment effects as well as 6- and 12-month follow-ups to assess long-term effects. Further, we will evaluate the interaction of these two factors in an explorative manner. A secondary aim of the trial is to evaluate changes between screening and treatment start, more specifically the immediate effects of the pre-treatment assessment interview on alcohol consumption and motivation to change. We hypothesize that those receiving a pre-treatment assessment interview will demonstrate greater alcohol reductions and greater motivation to change at the start of treatment compared to participants not receiving a pre-treatment assessment interview. We further hypothesise that these pre-treatment reductions will be significantly associated with drinking outcomes at post-treatment and at subsequent follow-ups.

## Methods/design

### Study design

This study is a 2X2 factorial superiority RCT where participants will receive access to the Alcohol Change Course, an eight-week ICBT program, and either a pre-treatment assessment interview (Factor 1), guidance from a health educator (Factor 2), a combination of these, or none of these. The study has been registered at www.clinicaltrials.gov (NCT03984786), and was approved by the University of Regina Ethics Review Board (approval number 2019–058).

### Setting

The trial is being conducted in the Online Therapy Unit (OTU; www.onlinetherapyuser.ca), based at the University of Regina, Saskatchewan, Canada, a clinic that has been operating since 2010 and routinely offers ICBT to residents of Saskatchewan free of charge. The OTU is financed by the Saskatchewan Ministry of Health. As of October 2019, the unit has worked with over 6000 clients and currently offers ICBT for depression and anxiety, chronic pain and other chronic conditions, and now alcohol misuse.

### Recruitment and procedure

The trial is being advertised online across Canada through the use of ads on Google and Facebook. Further, advertising emails, posters and flyers are being sent out to front-line service providers in Saskatchewan, Canada as well as to key organizations across Canada who commonly come in contact with people with alcohol problems. Potential participants who click on the link in the online ad, or who visit the website stated on the poster/flyer, are taken to a webpage with information about the course and an online screening form. The online screening form provides detailed information about the study and about the OTU after which potential participants are asked to fill out a consent form. Potential participants are then assessed for eligibility through a survey covering demographic information (e.g., sex, ethnicity, location), contact details (e.g., telephone number, email address), information about alcohol, depression and anxiety, and relevant background information (e.g., medical history, mental health history, symptoms). The survey system used in this study is encrypted and password-protected. Upon completing the survey, potential participants are to book a telephone enrollment call with unit staff through an online appointment booking software. In the telephone enrollment call, conducted in close proximity to the online screening (within 1–2 weeks), potential participants are asked a series of follow-up questions to the screening questionnaire by unit staff to ensure eligibility as noted below, and are also once again asked to consent to participation.

### Inclusion and exclusion criteria

To be included in the trial, participants must a) be 18 years or older; b) be a Canadian resident; c) have access to the internet, d) score 8 or more on the Alcohol Use Disorder Identification Test (AUDIT) [35] and e) have consumed 14 standard drinks or more in the preceding week. Participants are excluded from the trial if they present with a) severe depression defined as > 24 on the Patient Health Questionnaire PHQ-9 [36], b) risk of suicide defined as > 2 on suicidal ideation item of PHQ-9 [36], c) other severe mental health or medical conditions (e.g. unmanaged bipolar disorder, schizophrenia etc), d) severe substance use problems other than alcohol defined as > 24 on the Drug Use Disorder Identification Test (DUDIT) [37] or as assessed in the telephone enrollment call, e) low motivation to engage with online treatment as assessed in the telephone enrollment call, f) ongoing or impending significant mental health treatment defined as seeing a mental health professional more frequently than twice a month or g) past year hospitalization for mental health reasons. Applicants excluded from the trial are either referred to more appropriate mental health services in their area, or offered the Alcohol Change Course without being included in the trial.

#### Randomization

**Fig. 1:**
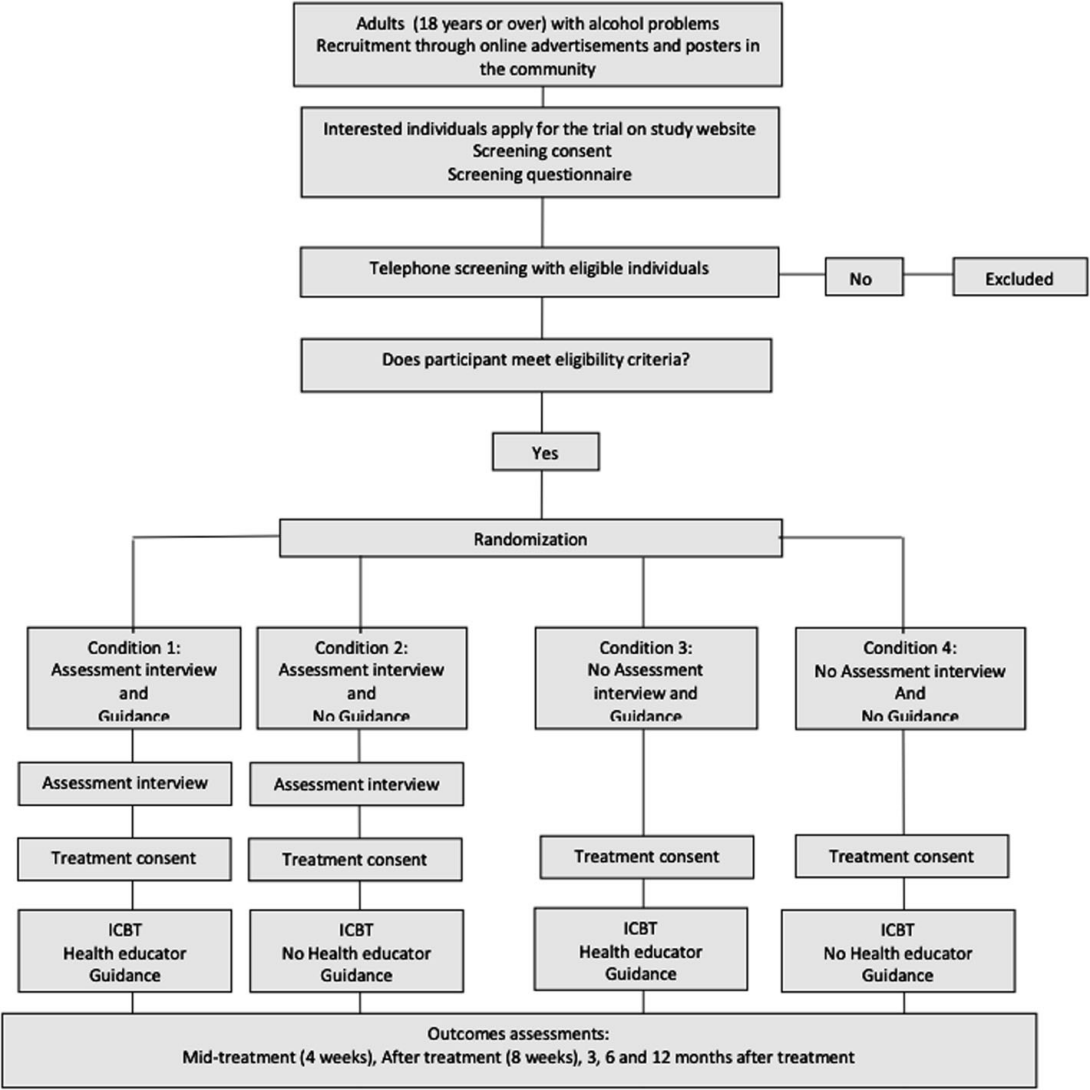
Flow of participants in the study

The unit staff are conducting the randomization during the telephone enrollment call as soon as inclusion criteria have been verified and the participant has been included. Randomization is in blocks of 16 to one of four conditions. The randomization scheme used in this trial was generated from the website http://www.randomization.com by author CS and uploaded to the survey system used for data collection in this trial. The randomization scheme is hidden from the staff person randomizing. All participants are blinded to the factors investigated. See Fig. 1 for participant flow throughout the study.

### Main intervention: the Alcohol Change Course

**Fig. 2:**
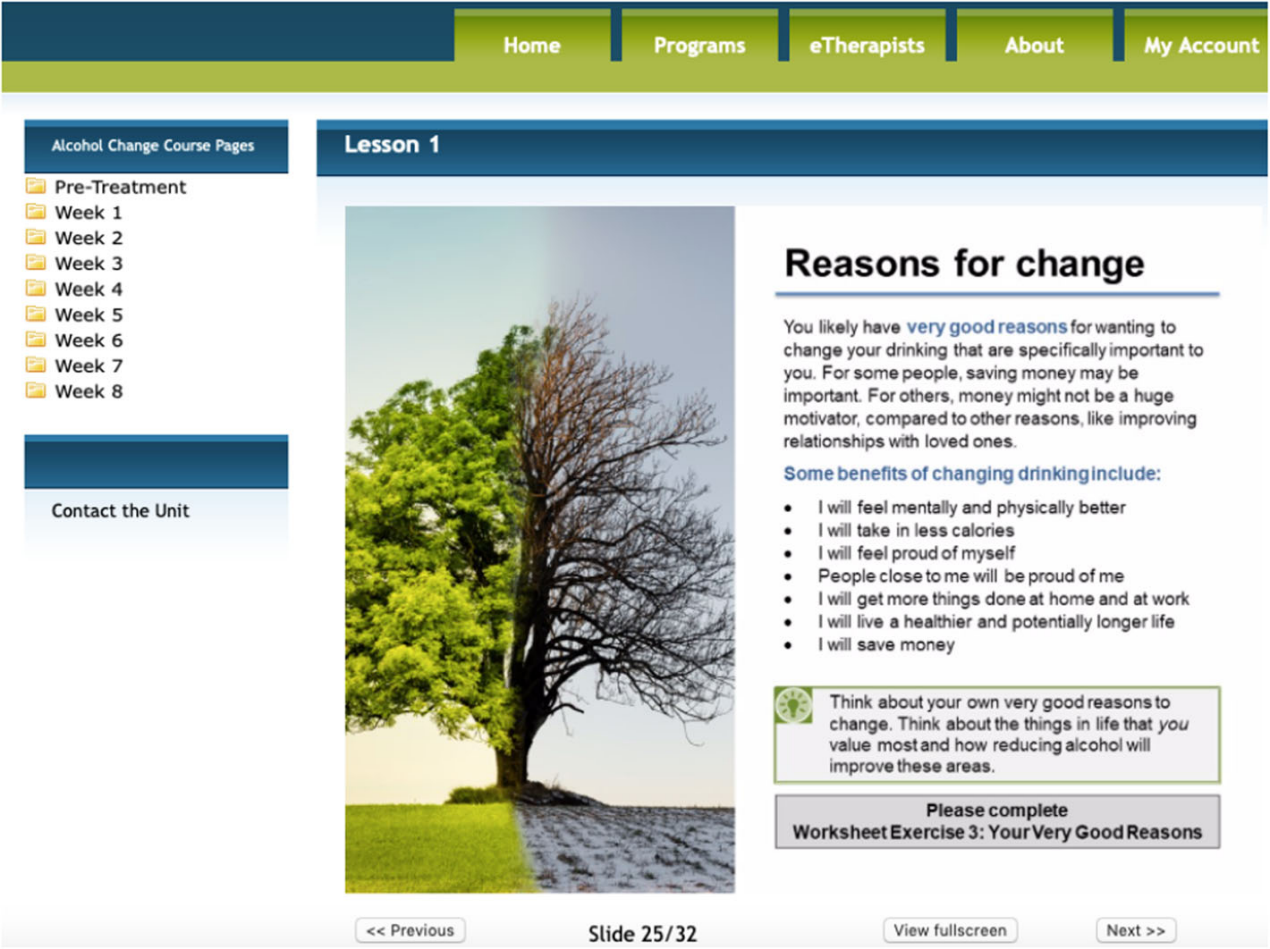
Screenshot of a page from Lesson 1 in the Alcohol Change Course

The Alcohol Change Course is an ICBT program targeting alcohol misuse (see Table 1 and Fig. 2). It comprises 12 online lessons (participants are granted access to 1 to 2 lessons per week over 8 weeks), and the content is based on CBT and relapse prevention [15]. The program was originally developed by researchers in Switzerland and is currently being used in a trial in Canada aimed at young adults [38]. For the current study, the program was adapted to conform to an adult population and to the format used in other programs at the OTU. Materials in courses provided at the OTU are all presented in a didactic (i.e., text-based with visual images) and case-enhanced learning format (i.e., educational stories demonstrating the application of skills). In addition to these changes, information about drinking guidelines in Canada, about abstinence and about the effects of alcohol on the body were added to the first lesson of the program. Further, all clients in the version provided at the OTU are provided worksheets at the end of each lesson with quizzes and exercises pertaining to the lesson. These worksheets can be downloaded and retained longer term by the client. The course is provided on the platform used at the OTU, which is encrypted and password-protected.

**Table 1:** Summary of module content in the Alcohol Change Course

**Week 1**	
**Lesson 1: Introduction to the Alcohol Change Course** Education about alcohol and goal setting regarding the client’s drinking for the duration of the course.	
**Lesson 2: Strategies for meeting your goals** Provision of simple strategies to help the client stick to their chosen drinking goal.	
**Week 2**	
**Lesson 3: Identifying risk situations** Prompts client to consider in which situations they tend to drink, and addresses how to prepare for these situations.	
**Lesson 4: Say yes to positive activities** Emphasizes the importance of engaging in positive activities that do not involve alcohol and how to effectively integrate these activities into the client’s daily life.	
**Week 3**	
**Lesson 5: Learning to say no to alcohol** Practice refusing to drink alcohol when it is offered and discussion of what situations or beliefs might make saying no particularly difficult.	
**Lesson 6: Coping with cravings** Learning about cravings for alcohol; how they feel for you, what causes them, and how to effectively deal with them.	
**Week 4**	
**Lesson 7: Problem solving** Introduces a plan to deal with stress-inducing situations, as a way to reduce the risk of turning to drinking to cope.	
**Week 5**	
**Lesson 8: Challenge your thought patterns!** Learning how to identify and challenge negative thoughts and how these may be related to drinking.	
**Week 6**	
**Lesson 9: Meeting your needs** A reminder about things that have a big impact on drinking behaviours; sleep, worry and anxiety, and social connections.	
**Lesson 10: Progressive Muscle Relaxation** Relaxation exercise that many people find helpful, especially when they experience craving.	
**Week 7**	
**Lesson 11: Dealing with slips and relapses** Provides definitions of slips and relapses, what may have caused them and what to do if they happen. The client is offered to write a final relapse plan.	
**Week 8**	
**Lesson 12: Preserve your success** Reviews the key messages of the course and helps the client think about how to maintain their successes and improvements in their drinking after the course has ended.

#### Monitoring of participants during the course

Each week throughout the 8-week course, all participants who log in to the platform will complete two questions about past week alcohol use; 1) How many drinks have you had in the past week? and 2) Over how many days did you consume these drinks? They will also complete the Patient Health Questionnaire-4 (PHQ-4), a brief questionnaire assessing depression and anxiety [39] as well as item 9 from PHQ-9 assessing suicidal ideation. Finally, they will also respond to some homework reflection questions. These weekly questionnaires are not intended as outcome measures, but are included to allow health educators and researchers to systematically monitor client symptoms as a safety measure during treatment.

### Experimental factors

The two experimental factors in the trial are:

Factor 1: Pre-treatment assessment interview.

Factor 2: Guidance from a health educator.

Thus, participants are being randomized to one of the following four conditions:

Condition 1: Pre-treatment assessment interview and health educator guidance.

Condition 2: Pre-treatment assessment interview and no health educator guidance.

Condition 3: No pre-treatment assessment interview and health educator guidance.

Condition 4: No pre-treatment assessment interview and no health educator guidance.

### Factor 1: pre-treatment assessment interview

Participants randomized to the pre-treatment assessment interview (conditions 1 and 2) receive the interview immediately following randomization in the enrollment telephone call. In the pre-treatment assessment interview, the staff person explores the participant’s alcohol habits in a supportive manner using the AUD module in the Structured Clinical Interview Diagnostic Statistical Manual 5 - Research Version (SCID-5 RV) [40], i.e. first asking general questions about the participant’s alcohol use over the past year, followed by more specific questions pertaining to each of the eleven AUD criteria of the DSM-5 as they relate to the participant. The main purpose of the interview is not to establish a diagnosis, but to allow the participant to verbalize their alcohol issues together with the staff person. Therefore, the number of positive criteria is not reflected back to the participant. The assessment interview takes about 20–30 min in addition to the telephone enrollment call. At the end of the assessment interview, the participant is provided a username and temporary password, and informed that they can log in to the Alcohol Change Course on the second Monday following the randomization date. Participants randomized to no assessment interview (conditions 3 and 4) are provided the username and temporary password immediately after the randomization, after which the telephone enrollment call ends. Similarly, these participants gain access to the Alcohol Change Course on the second Monday from the randomization date. The purpose of having all participants access the Alcohol Change Course on the second Monday after randomization instead of immediately following randomization is that this allows for a pre-treatment phase (time between screening interview and start of treatment) of between 10 and 20 days for all four groups, permitting evaluation of pre-treatment changes related to the assessment interview.

### Factor 2: health educator guidance

Participants randomized to receive health educator guidance (conditions 1 and 3) have access to health educator guidance during the eight-weeks of the course. Guides consist of social workers, counsellors and PhD psychology graduate students under supervision, with past experience delivering ICBT. The health educator’s job is to answer participant’s questions, reinforce module completion and boost motivation. All written communication between participants and health educators takes place through a secure message system on the www.onlinetherapyuser.ca website, but participants may also be contacted by phone. Phone calls are made when participants request such contact or the health educator feels a phone call would assist with client care (e.g., assess risk, address misunderstanding). When phone calls are made, health educators write a progress note on the participant’s record specifically noting the date/time of the call, and nature of contact. Health educators are spending approximately 15 min per week and participant. Health educators are blinded to whether participants have received a pre-treatment assessment interview or not.

All participants receive automated messages about the course that are sent to their regular email. These emails mainly inform the participant about any new modules accessible on the website platform. Those randomized to no guidance from a health educator (conditions 2 and 4) only receive these weekly automated email messages. They are also able to contact the OTU regarding any technical issues they experience with the site. Furthermore, a member of the research team reviews participant responses to weekly measures on alcohol consumption and depression/anxiety. Participants in conditions 2 and 4 are only contacted by the research team if the weekly measures indicate a significant clinical issue requiring attention, e.g., major increase in alcohol consumption (assessed on a case-by-case basis), a sudden increase of depression symptoms (defined as an increase of 5 in PHQ-4 since previous week) or suicidal ideation (defined as > 2 on item 9 from PHQ-9). If any participant is found to be at risk, they will be contacted and referred to appropriate health care. However, they will only be discontinued from the intervention and trial if they request it.

### Measures

All participants are asked to complete online measures at screening, pre-treatment, mid-treatment (4 weeks into the treatment), post-treatment (8 weeks) and at 3, 6 and 12 months post-treatment. Participants who do not complete these questionnaires are contacted via telephone and/or email to remind them to complete the measures. Participants receive a maximum of three reminders per follow-up period.

The primary outcome of the trial will be alcohol consumption in the past week as measured by Time Line Follow Back [41]. This outcome will be calculated in two ways: number of drinks in preceding week and number of heavy drinking days (defined as 4 or more drinks per day for women and 5 or more drinks per day for men) in preceding week. Secondary measures include alcohol-related problems as measured by AUDIT [35] which is a 10-item instrument. Alcohol craving will be measured by Penn Alcohol Craving Scale (PACS) [42], an instrument consisting of five items found to have both high internal consistency as well as high predictive, convergent and discriminatory validity. Alcohol-related self-efficacy will be measured by the Brief Situational Confidence Questionnaire (BSCQ) [43], consisting of eight items derived from the relapse prevention model [15]. The instrument has been found to have high both internal consistency and concurrent validity [43]. Further, the Sheehan Disability Scale (SDS) [44] adapted to alcohol’s impact on daily functioning will be used, where reliability and validity is unknown. Depression will be measured by the Patient Health Questionnaire-9 (PHQ-9) [36] and anxiety will be measured by the Generalized Anxiety Disorder-7 (GAD-7) [45], both well validated instruments. The Readiness to Change questionnaire – Treatment Version (RCQ-TV) [46] will be used at screening and at pre-treatment to assess immediate motivational changes due to the assessment interview, and to assess relation to lont-term outcomes. We will also administer the Credibility/Expectancy Questionnaire [47] at mid-treatment to assess credibility of treatment. Lastly, at post-treatment, we will administer questions pertaining to treatment evaluation and negative effects. See Table 2 for a presentation of measures included at each time point in the study.

**Table 2:** Measures in the screening, pre-treatment, mid-treatment, post-treatment and 3, 6 and 12 month follow-ups in the Alcohol Change Course trial

Outcome Measure	Screening	Pre-Treatment	Mid-Treatment	Post-Treatment	3 Months	6 Months	1 Year
**Consent**	✓	✓					
**Alcohol consumption (TLFB)**	✓	✓	✓	✓	✓	✓	✓
**Depressive symptoms (PHQ-9)**	✓	✓	✓	✓	✓	✓	✓
**Alcohol problems (AUDIT)**	✓			✓	✓	✓	✓
**Anxiety (GAD-7)**	✓			✓	✓	✓	✓
**Alcohol craving (PACS)**	✓			✓	✓	✓	✓
**Alcohol-related self-efficacy (BSCQ)**	✓			✓	✓	✓	✓
**Readiness to change (RCQ-TV)**	✓	✓					
**Treatment Credibility& Expectancy**			✓				
**Evaluation & Negative Effects**				✓			

### Statistical analyses

The trial aims to recruit 300 participants to the four conditions (75 participants per group, see Table 3). We plan to use a multi-level hierarchical linear model with observations nested within participants when analyzing the outcome data as this method has been found superior in handling missing data in longitudinal designs. However, depending on the distribution of alcohol consumption outcomes, a multi-level hierarchical non-linear model or generalized estimating equations with an assumption of a binomial distribution may also be used. Analyses will follow the intent-to-treat principle, where all participants will be included for the outcome analysis. However, per-protocol analyses will also be conducted.

**Table 3:** Factorial trial design and target sample per group

		Factor 1: Pre-treatment assessment interview		Total
		Yes	No	
**Factor 2: Health educator guidance**	**Yes**	75	75	150
	**No**	75	75	150
**Total**		150	150	300

### Power calculation

To estimate the sample size, we used the Factorial Power Plan provided in the R package Multiphase Optimization Strategy. Regarding effect size estimates of factor 1 (assessment interview) we had no available studies to draw on. We therefore pragmatically decided to estimate the effect size as 0.35, as this was the minimum effect size that would indicate this factor to be worth implementing considering the time spent by staff conducting the interview. Regarding effect size estimates of factor 2 (guidance), we decided to estimate it as 0.35, based on findings in previously published studies [24, 25]. Power was set at 80% and we assumed a correlation of 0.5 between pre-and post-test measurements. We also assumed an attrition of 30%. This target sample size ensures that the study is powered to detect an effect of 0.30 in unique effects of either of the two factors.

### Ethical considerations

All participants receive detailed information about the study presented in writing prior to completing screening questionnaires and prior to starting the Alcohol Change course; furthermore, detailed information and consent are presented orally during the enrollment phone call. Participants receive detailed information regarding the nature and implications of their participation, potential risks and benefits of participation, the right to withdraw from the study at any time without consequences, and about confidentiality of information. Further, participants complete weekly questions about alcohol use, depression and anxiety allowing health educators and researchers to systematically monitor their symptoms, and in the case of a serious adverse event, such as suicide risk, we will contact emergency care.

### Timeline

Data collection began in July 2019 and is estimated to be completed by December 31, 2021.

## Discussion

Research suggests that the two factors investigated in this trial may contribute to clinical outcomes, however, they are both under-researched. Assessment reactivity is highly neglected in the alcohol literature, particularly in the form of experimental research [31]. To our knowledge, no previous studies on alcohol treatment have experimentally investigated the effects of a pre-treatment assessment interview, although there are observational studies suggesting that pre-treatment assessment interviews are associated with both immediate, short-term and long-term reductions in alcohol consumption [30, 34]. We have found one study investigating assessment reactivity in an internet brief intervention aimed at heavy drinking college students, where use of a delayed-assessment control group indicated the presence of assessment reactivity [48]. However, we have found no studies examining AR in studies on ICBT for alcohol misuse. As for guidance in ICBT for alcohol misuse, individual trials have found this factor to lead to greater alcohol reductions than ICBT without guidance [24, 25], but long-term outcomes are lacking and replication by new teams and in new settings is necessary to assess its clinical value.

The treatment gap for alcohol misuse remains the largest of all mental disorders, with only around 20% receiving treatment [5, 6] and identifying attractive and easily accessible avenues of care for this population remains an urgent clinical and public health matter. As ICBT has moved from mainly being a treatment form offered to participants in research studies to, in recent years, being implemented and integrated within existing health care services around the world [13], optimizing the delivery of ICBT becomes a natural focal point [49]. The factorial approach chosen in the current study allows for simultaneous investigation of two clinically important factors in optimizing the delivery of ICBT for people with alcohol misuse and results from the trial are expected to be of relevance to clinics and policy makers alike.

## Data Availability

Datasets generated in this trial will be considered upon request.

## Abbreviations

AUDIT: Alcohol Use Disorder Identification Test
BSCQ: Self Confidence Questionnaire
GAD-7: Generalized Anxiety Disorder 7
PACS: Penn alcohol craving scale
PHQ-9: Patient Health Questionnaire 9
RCQ (TV): Readiness to Change Questionnaire – Treatment Version
TLFB: Time Line Follow Back
